# HIV prevalence and risk behavior among male and female adults screened for enrolment into a vaccine preparedness study in Maputo, Mozambique

**DOI:** 10.1371/journal.pone.0221682

**Published:** 2019-09-17

**Authors:** Ivalda Macicame, Nilesh Bhatt, Raquel Matavele Chissumba, Leigh Anne Eller, Edna Viegas, Khelvon Araújo, Chiaka Nwoga, Qun Li, Mark Milazzo, Nancy K. Hills, Christina Lindan, Nelson L. Michael, Merlin L. Robb, Ilesh Jani, Christina S. Polyak

**Affiliations:** 1 Instituto Nacional de Saúde, Ministério da Saúde, Mozambique; 2 Military HIV Research Program, Walter Reed Army Institute of Research, Silver Spring, Maryland, United States of America; 3 Henry Jackson Foundation for the Advancement of Military Medicine, Bethesda, Maryland, United States of America; 4 University of California San Francisco, San Francisco, California, United States of America; Agencia de Salut Publica de Barcelona, SPAIN

## Abstract

**Introduction:**

Mozambique continues to have a significant burden of HIV. Developing strategies to control the HIV epidemic remains a key priority for the Mozambican public health community. The primary aim of this study was to determine HIV prevalence and risk behavior among males and females screened for a HIV vaccine preparedness study in Maputo, Mozambique.

**Methods:**

Male and female participants between 18–35 years old were recruited from the general community and from female sex worker (FSW) and lesbian, gay, bisexual, and transgender (LGBT) associations in Maputo. All participants were screened for HIV and a questionnaire was administered to each participant to assess HIV risk behavior.

**Results:**

A total of 1125 adults were screened for HIV infection, among whom 506 (45%) were male. Among men, 5.7% reported having had sex with men (MSM) and 12% of female participants reported having exchanged sex for money, goods or favors in the past 3 months. The overall HIV prevalence was 10.4%; 10.7% of women, and 10.1% of men were HIV infected; 41.4% of MSM were seropositive. HIV infection was associated with older age (25–35 years old) (OR: 6.13, 95% CI: 3.01, 12.5), MSM (OR: 9.07, 95% CI: 3.85, 21.4), self-perception of being at high-risk for HIV (OR: 3.99, 95% CI: 1.27, 12.5) and self-report of a history of a diagnosis of sexually transmitted infection (OR: 3.75, 95% CI: 1.57, 8.98).

**Conclusion:**

In our cohort, HIV prevalence was much higher among MSM compared to the overall prevalence. Behavioral factors were found to be more associated with HIV prevalence than demographic factors. The study findings demonstrate the critical importance of directing services to minority communities, such as MSM, when prevention strategies are being devised for the general population.

## Introduction

Worldwide, 36.9 million people are living with HIV infection and approximately half of them do not know their HIV status [1]. Despite a 41% drop in new HIV infections in sub-Saharan Africa since 2000, there were an estimated 1.4 million new infections reported in 2014, representing 67% of the total number of new infections globally [1]. Mozambique is among the 10 countries with the highest HIV burden in the world, with a HIV prevalence of 13.2% in adults aged 15 to 49 years [2]. Maputo City, the capital and largest city of Mozambique, has an even higher prevalence with 16.9% of the general population estimated to be infected [2]. In key populations, female sex workers (FSW) are at particularly high-risk, with 31.2% HIV infected [3]; among MSM the prevalence was 8.2% [4].

Similar to other African countries, Mozambique has introduced several strategies for the prevention of HIV. HIV vaccines have shown to be cost-effective under conditions related to their efficacy, price and HIV incidence in the target population [5–7]. Thus, a network of clinical trial sites has been established to expeditiously conduct exploratory and early phase development studies and support the eventual conduct of HIV vaccine efficacy trials in African countries. Cohort development is an important component of this strategy as higher risk populations are key to these future trials. As part of its involvement in the conduct of HIV vaccine trials, *Instituto Nacional de Saúde* (INS) in Mozambique established a cohort of low risk youths (18 to 24 years old) in Maputo City, who participated in a phase I vaccine trial [8]; the HIV prevalence at baseline was 5.1% [9]. In order to prepare Mozambique to implement a phase III vaccine trial, we initiated a cohort and site development study to assess the incidence of HIV infection, retention rate, and willingness to participate in future HIV vaccines trials. Here, we describe HIV prevalence and factors associated with HIV infection at screening among those recruited into this longitudinal observational cohort.

## Materials and methods

### Study population

From November 2013 to November 2014, we recruited 18–35 year old male and female residents of Maputo City using a community-based recruitment strategy. Trained study staff distributed fliers at multiple urban and peri-urban sites, including night schools, bars, and markets of Maputo city. The recruitment staff was composed by two (2) social scientists (one female and one male), three (3) hired recruitment staff (one female, one male and one transgender), five (5) recruiters from the local lesbian, gay, bisexual and transgender (LGBT) associations, two (2) female sex workers (FSW), eleven (11) staff from local community-based organizations focused on HIV, ten (10) staff from health facilities and youth clinics and six (6) residents from Polana Caniço neighborhood. Fliers indicating that a study was being conducted among adults who did not know their HIV status were distributed by the recruitment staff every day (day and night) during the course of one year. Persons who were interested were directed to the study site, a research center (*Centro de Investigação e Treino em Saúde da Polana Caniço*–CISPOC) affiliated to INS located in a peri-urban area of the city, to learn more about the study and be screened for eligibility.

Study staff explained details of the study to potential participants who presented for screening. Those who were willing to be screened signed an informed consent form and were required to successfully complete a competency test to ensure understanding of study procedures. Volunteers were allowed three attempts on the competency test to achieve a passing score of 80%. Consented participants completed an interviewer-administered questionnaire. The questionnaire included information about socio-demographics; age at first sex; sexual risk behavior in the last three months (total number of partners, presence of a primary partner defined as a boyfriend, girlfriend or spouse, and presence of a secondary partner); frequency of condom use with primary and secondary partners in the last three months; engaging in transactional sex, defined as exchanging goods or money for sex in the last three months; alcohol use (proportion of times that alcohol was used during sex in the last three months); history of recreational drug use; circumcision among males; and self-reported history of being diagnosed with a sexually transmitted infection (STI) by a health professional in last three months. Participants also underwent a clinical evaluation and were screened for STIs (syphilis laboratory testing and a clinical non-laboratory diagnosis based on clinical inspection and history for other STIs, according to the national guidelines [10]). Treatment was provided, based on national guidelines. Uncircumcised male participants underwent counseling regarding circumcision and were referred for voluntary medical circumcision. After the approximately three (3) hours visit (which includes the consent process, questionnaires, laboratory testing and disclosure of the HIV result), all participants who consented and performed the study procedures received 150 Meticais (approximately 2.50 USD), a bottle of water, and condoms as compensation for time and transportation. This study was approved by the National Health Bioethics Committee of Mozambique and by the Walter Reed Army Institute of Research in Silver Spring, Maryland.

### Laboratory testing

Syphilis and rapid HIV tests were performed on site during the screening visit. Those who had evidence of syphilis and/or were HIV infected were referred to a public health clinic for treatment and care.

Venous blood samples were screened for HIV antibodies by using the rapid test Alere Determine^®^ HIV-1/2 (Alere, Japan). Reactive samples were confirmed by a second rapid test, the Unigold HIV 1/2^®^ (Trinity Biotech PLC, Ireland). Indeterminate and discordant results were resolved by using a fourth-generation ELISA, Genscreen Ultra HIV Ag-Ab^®^ (Biorad, France).

#### Syphilis test

The diagnosis of syphilis was performed on batched samples. Briefly, serum samples were screened for syphilis by using a rapid plasmin reagin (RPR) test (Human Diagnostics Worldwide, Germany). Samples reactive at any titer were evaluated with a treponemal specific test, Serodia^*®*^-TP-PA (Fujirebio, Japan). Samples positive by both RPR and TPPA were considered positive for syphilis.

### Statistical analysis

Data were double-entered into ClinPlus software (Bound Brook, NJ, USA) and exported to Stata version 14 (StataCorp LLC, College Station, Texas, USA) for analysis. Cohort characteristics were summarized using frequencies and proportions for categorical data. Cross tabulations are supplied for categorical variables and tested using chi-squared or Fisher’s exact test, where appropriate. For continuous variables, means and standard deviations were utilized for normally distributed data and differences were assessed using Student’s t-test. Medians and interquartile ranges were utilized for data that were not normally distributed and differences between groups were tested using the Wilcoxon rank sum test. These characteristics were summarized for the entire cohort as well as stratified by gender. Factors associated with testing positive for HIV at screening were summarized similarly for continuous and categorical variables, and then were stratified by HIV status. Univariate logistic regression analyses were conducted to examine characteristics potentially associated with the presence of HIV infection at screening. Results are presented as odds ratios with 95% confidence intervals and p-values for the association between HIV positivity and characteristics. Variables significant at α = 0.05 level were included in multivariable analysis. Because sexual behavior characteristics were frequently correlated, we retained in the model those variables for which the least amount of data were missing. Our final multivariable model was selected by using likelihood ratio testing to compare nested models. When a model containing a variable did not differ significantly from a model which did not include the variable, the variable was dropped from the model. After choosing a final model, specification of the model was tested using a link test. Goodness-of-fit was determined using the Hosmer-Lemeshow test, and tests of multicollinearity, influence, and leverage were used to test for the appropriate inclusion of individual observations.

## Results

From November 2013 to November 2014, more than 3000 fliers were distributed to the community and 1150 participants were screened for the study. Among those screened, 25 were excluded from analysis due to the lack of an HIV test result at screening resulting in a total of 1125 participants included in this analysis. The baseline socio-demographic, clinical, and behavioral characteristics of these 1125 participants are presented in Table 1. The mean age was 22.5years with a standard deviation of 4.2, 55% were women and more than three quarters (77%) of the participants were single. The majority of the participants (88.3%) had at least some secondary education and more than half (53%) were full-time students. Mean age at sexual debut was 16.7 years old. The median number of sexual partners in the last three months was 2, with women reporting fewer sexual partners than men (45% vs. 28.4% reporting fewer than two sexual partners in the last three months, respectively). Of those who reported only a primary partner, 12.2% (116/953) reported consistent condom use. Of those who reported a primary and a secondary partner, 29.9% (86/228) reported consistent condom use. A total of 5.7% (29/506) of male participants reported having sex with men, and 12% (71/590) of female participants reported exchanging sex for money, goods or favors in the last three months. Fewer than one-quarter (21.1%) of the females exchanging sex for money, goods or favors reported consistent condom use with those partners. Only 18 participants (17 men) reported a history of non-injectable recreational drug use and no participants reported a history of injecting drugs. More women (5.0%) than men (2.2%) reported having received a diagnosis of an STI in the last three months; however, more men (4.2%) were diagnosed with syphilis compared to women (1.6%) at baseline. Less than two-thirds (58.1%) of the men had been circumcised.

**Table 1:** Baseline socio-demographic and behavioral characteristics of adults screened from 2013–2014 for enrolment in a vaccine preparedness trial, Maputo city, Mozambique (N = 1125).

Characteristics		Total (N = 1125)		Male (n = 506)		Female (n = 619)	
		N	%	n	%	n	%
Age, yrs; mean (sd)		22.5	(4.2)	22.6	(4.0)	22.4	(4.3)
Age, yrs							
	18–20	456	(40.5)	188	(37.2)	268	(43.3)
	21–24	381	(33.9)	185	(36.6)	196	(31.7)
	25–35	288	(25.6)	133	(26.3)	155	(25.0)
Marital status							
	Single	866	(77.0)	412	(81.4)	454	(73.3)
	Married/ Cohabiting	211	(18.8)	81	(16.0)	130	(21.0)
	Separated/ Widowed	47	(4.2)	13	(2.6)	34	(5.5)
Education							
	Primary or lower	132	(11.7)	71	(14.0)	61	(9.9)
	Secondary or higher	993	(88.3)	435	(86.0)	558	(90.1)
Employment							
	Unemployed	152	(13.5)	59	(11.7)	93	(15.0)
	Housewife/ househusband	31	(2.8)	4	(0.8)	27	(4.4)
	Full-time Student	596	(53.0)	223	(44.1)	373	(60.3)
	Employed	346	(30.8)	220	(43.5)	126	(20.4)
Monthly income (N = 498)*							
	None	157	(31.5)	65	(23.3)	92	(42.0)
	5000 or less	252	(50.6)	155	(55.6)	97	(44.3)
	> 5000	89	(17.9)	59	(21.1)	30	(13.7)
Age at sexual debut, yrs; mean (sd)		16.7	(1.9)	16.6	(2.1)	16.8	(1.7)
Age at sexual debut, yrs				504		618	
	<15	146	(13.0)	82	(16.3)	64	(10.4)
	15–18	790	(70.2)	308	(61.1)	482	(78.0)
	19–24	132	(11.7)	64	(12.7)	68	(11.0)
	≥25	54	(4.8)	50	(9.9)	4	(0.6)
No. sex partners, last 3 mos; median (IQR)		2	(1.2)	2	(1.3)	2	(1.2)
No. sex partners, last 3 mos,							
	1	399	(37.6)	134	(28.4)	265	(45.0)
	2	410	(38.6)	197	(41.7)	213	(36.2)
	≥3	252	(23.8)	141	(29.9)	111	(18.8)
Primary sex partner		1062		472		590	
	Yes	953	(89.7)	407	(86.2)	546	(92.5)
	No	109	(10.3)	65	(13.8)	44	(7.5)
Condom use with primary sex partner		953		407		546	
	Never	81	(8.5)	35	(8.6)	46	(8.4)
	Sometimes	756	(79.3)	313	(76.9)	443	(81.1)
	Always	116	(12.2)	59	(14.5)	57	(10.4)
Secondary sex partner		1062		472		590	
	Yes	288	(27.1)	144	(30.5)	144	(24.4)
	No	774	(72.9)	328	(69.5)	446	(75.6)
Condom use with secondary sex partner		288		144		144	
	Never	16	(5.6)	9	(6.3)	7	(4.9)
	Sometimes	186	(64.6)	89	(61.8)	97	(67.4)
	Always	86	(29.9)	46	(31.9)	40	(27.8)
Male-male sex, last 3 mos				29	(5.7)	n/a	
Exchange sex for goods/money, last 3 mos		1062		472		590	
	Yes	84	(7.9)	13	(2.8)	71	(12.0)
	No	978	(92.1)	459	(97.2)	519	(88.0)
Frequency of exchanging sex, last 3 mos							
	Never	0		0		0	
	Sometimes	56	(66.7)	10	(76.9)	46	(64.8)
	Always	28	(33.3)	3	(23.1)	25	(35.2)
Frequency of condom use during transactional sex		(N = 84)		(N = 13)		(N = 71)	
	Never	4	(4.8)	0		4	(5.6)
	Sometimes	57	(67.9)	5	(38.5)	52	(73.2)
	Always	23	(27.4)	8	(61.5)	15	(21.1)
Self-perceived HIV risk		1107		499		608	
	No risk	176	(15.9)	71	(14.2)	105	(17.3)
	Some risk	858	(77.5)	399	(80.0)	459	(75.5)
	High-risk	60	(5.4)	26	(5.2)	34	(5.6)
	I am HIV+	13	(1.2)	3	(0.6)	10	(1.6)
Sex after drinking, last 3 months		1058		468		590	
	Never	528	(49.9)	191	(40.8)	337	(57.1)
	Sometimes	516	(48.8)	268	(57.3)	248	(42.0)
	Always	14	(1.3)	9	(1.9)	5	(0.8)
History of non-injection drug use		1124		505		619	
	Yes	18	(1.6)	17	(3.4)	1	(0.2)
	No	1106	(98.4)	488	(96.6)	618	(99.8)
Self-report of STI diagnosis, last 3 mos		1121		502		619	
	Yes	42	(3.7)	11	(2.2)	31	(5.0)
	No	1079	(96.3)	491	(97.8)	588	(95.0)
Syphilis test							
	Positive	31	(2.8)	21	(4.1)	10	(1.6)
	Negative	1094	(97.2)	486	(95.9)	609	(98.4)
Circumcised							
	Yes			294	(58.1)	n/a	
	No			212	(41.9)		

*Monthly income is presented only among those who were not housewives/househusbands or students.

Mos–month

Yrs–years

The overall HIV prevalence among the screened participants was 10.4%, with no differences between women and men (Table 2). In bivariate analyses, being older (25–35 years old) [odds ratio (OR): 8.5, 95% confidence interval (CI): 4.82, 15.0] and being separated or widowed (OR: 4.09, 95% CI: 2.07, 8.09) were significantly associated with testing positive for HIV. Those who reported having known HIV seropositive sexual partners were almost five times as likely to be HIV positive (OR: 4.74, 95% CI: 2.16, 10.4). Those who never used condoms with primary and secondary sexual partners had 4 (OR: 4.16, 95% CI: 1.79, 9.66) and 8 (OR: 8, 95% CI: 2.16, 29.6) times the odds of being HIV seropositive, respectively. Among men who had sex with men (MSM), 41.4% were HIV infected (95% CI: 0.235, 0.611); MSM were more likely to be HIV seropositive (OR: 6.66, 95% CI: 3.10, 14.3) than men who were strictly heterosexual. Reporting transactional sex was not associated with HIV infection. Those who felt that they were at high-risk of HIV were more likely to be HIV infected (OR: 4.26, 95% CI: 1.51, 12). Participants with a previous diagnosis of STI were 2.45 (95% CI: 1.14–5.26) times more likely to be HIV positive than those not reporting having an STI in the last three months.

**Table 2:** Risk factors associated with HIV infection among adults screened from 2013–2014 for enrolment in a vaccine preparedness trial, Maputo city, Mozambique (N = 1125).

Characteristics		Total	HIV positive		Unadjusted			Adjusted		
		N	n	%	OR	95% CI	p-value	OR	95% CI	p-value
Total		1125	117	(10.4)						
Sex										
	Male	506	51	(10.1)	Ref					
	Female	619	66	(10.7)	1.06	(0.72, 1.57)	0.75			
Age in years; mean (sd)		22.5 (4.2)	25.8	(4.7)	1.19	(1.15, 1.24)	<0.0001			
Age in years, categorized										
	18–20	456	16	(3.5)	Ref			Ref		
	21–24	381	33	(8.7)	2.61	(1.41, 4.82)	0.002	1.91	(0.96, 3.80)	0.07
	25–35	288	68	(23.6)	8.50	(4.82, 15.0)	<0.0001	6.13	(3.01, 12.5)	<0.0001
Marital status										
	Single	866	74	(8.5)	Ref			Ref		
	Married/ Cohabiting	211	30	(14.2)	1.77	(1.13, 2.79)	0.01	0.55	(0.30, 0.99)	0.046
	Separated/ Widowed	47	13	(27.7)	4.09	(2.07, 8.09)	<0.0001	0.99	(0.41, 2.38)	0.98
Education										
	Primary or lower	132	37	(28.0)	Ref			Ref		
	Secondary or higher	993	80	(8.1)	0.22	(0.14, 0.35)	<0.0001	0.37	(0.21, 0.65)	0.001
Employment										
	Unemployed	152	25	(16.4)	Ref			Ref		
	Housewife/ househusband	31	3	(9.7)	0.54	(0.15, 1.93)	0.35	0.69	(0.18, 2.72)	0.60
	Full-time Student	596	30	(5.0)	0.27	(0.15, 0.47)	<0.0001	0.46	(0.23, 0.91)	0.03
	Employed	346	59	(17.1)	1.04	(0.63, 1.74)	0.87	0.69	(0.18, 2.72)	0.23
Monthly income, MZN										
	None	157	25	(15.9)	Ref					
	5000 or less	252	45	(17.9)	1.15	(0.67, 1.96)	0.61			
	> 5000	89	14	(15.7)	0.99	(0.48, 2.01)	0.97			
Age, sexual debut, yrs; mean (sd)		16.7 (1.9)	16.7	(2.2)	0.99	(0.89, 1.10)	0.88			
Age, sexual debut, yrs										
	<15	146	20	(13.7)	Ref					
	15–18	790	75	(9.5)	0.66	(0.39, 1.12)	0.12			
	19–24	132	14	(10.6)	0.75	(0.36, 1.55)	0.43			
	≥25	54	8	(14.8)	1.10	(0.45, 2.66)	0.84			
No. sex partners, last 3 mos, median (IQR)		2 (1, 2)	2	(1.2)	1.02	(0.98, 1.06)	0.36			
No. sex partners, last 3 mos,										
	1	399	43	(10.8)	Ref					
	2	410	41	(10.0)	0.92	(0.59, 1.45)	0.72			
	≥3	252	27	(10.7)	0.99	(0.60, 1.65)	0.98			
Primary sex partner										
	No	109	14	(12.8)	1.29	(0.71, 2.34)	0.71			
	Yes	953	97	(10.2)	Ref					
	Missing	87	6	(6.9)						
Condom use, primary sex partner										
	Never	81	21	(25.9)	4.16	(1.79, 9.66)	0.001			
	Sometimes	756	67	(8.9)	1.16	(0.56, 2.39)	0.70			
	Always	116	9	(7.8)	Ref					
Secondary sex partner										
	No	774	84	(10.9)	Ref					
	Yes	288	27	(9.4)	0,85	(0.54, 1.34)	0.48			
Condom use, second. sex partner										
	Always	86	6	(7.0)	Ref					
	Sometimes	186	15	(8.1)	1.17	(0.44, 3.13)	0.76			
	Never	16	6	(37.5)	8.00	(2.16, 29.6)	0.002			
Known HIV + sex partners										
	No	1006	96	(9.5)	Ref					
	Yes	30	10	(33.3)	4.74	(2.16, 10.4)	<0.0001			
No. HIV+ sex partners,last 3 mos										
	None	1026	98	(9.6)	Ref					
	1 partner	22	10	(45.5)	4.74	(2.16, 10.4)	<0.0001			
	2 partners	1	0		n/a					
	3–5 partners	0	0		n/a					
	More than 5 partners	0	0		n/a					
Condom use with HIV+ sex partner										
	Never	7	2	(28.6)	0.39	(0.06, 2.70)	0.35			
	Sometimes	16	8	(50.0)	Ref					
	Always	0	0							
Sex with a partner ≥ 10 years younger, last 3 mos										
	No	1040	107	(10.3)	Ref					
	Yes	21	4	(19.0)	2.05	(0.68, 6.21)	0.20			
Sex with a partner ≥ 10 years older, last 3 mos										
	No	1019	102	(10.0)	Ref					
	Yes	40	9	(22.5)	2.61	(1.21, 5.64)	0.02			
Male-male sex, last 3 mos		29	12	(41.4)	6.66	(3.10, 14.3)	<0.0001	9.07	(3.85, 21.4)	<0.0001
Exchange sex for goods/money, last 3 mos										
	No	978	99	(10.1)	Ref					
	Yes	84	12	(14.3)	1.48	(0.78, 2.82)	0.23			
Frequency of exchanging sex, last 3 mos^a^										
	Never	0	0							
	Sometimes	56	6	(10.7)	Ref					
	Always	28	6	(21.4)	2.27	(0.66, 7.84)	0.19			
Frequency of condom use during transactional sex										
	Never	4	2	(50.0)	4.75	(0.51, 44.5)	0.17			
	Sometimes	57	6	(10.5)	0.56	(0.14, 2.20)	0.41			
	Always	23	4	(17.4)	Ref					
Self-perceived HIV risk										
	No risk	176	7	(4.0)	Ref			Ref		
	Some risk	858	85	(9.9)	2.65	(1.21, 5.84)	0.02	2.72	(1.18, 6.29)	0.02
	High-risk	60	9	(15.0)	4.26	(1.51, 12.0)	0.006	3.99	(1.27, 12.5)	0.02
	I am HIV+	13	13	(100.0)	n/a					
Sex after drinking, last 3 mos										
	Never	528	41	(7.8)	Ref					
	Sometimes	516	67	(13.0)	1.77	(1.18, 2.67)	0.006			
	Always	14	3	(21.4)	3.24	(0.87, 12.1)	0.08			
History of non-injectable drug use										
	No	1106	113	(10.2)	Ref					
	Yes	18	4	(22.2)	2.51	(0.81, 7.76)	0.11			
Self-report of STI diagnosis, last 3 mos										
	No	1079	108	(10.0)	Ref			Ref		
	Yes	42	9	(21.4)	2.45	(1.14, 5.26)	0.02	3.75	(1.57, 8.97)	0.003
Tested positive for syphilis		31	3	(9.7)	0.92	(0.28, 3.08)	0.89			
Circumcised (man only)										
	No	212	39	(18.4)	Ref					
	Yes	294	12	(4.1)	0.19	(0.10, 0.37)	<0.0001			

All p-values calculated using chi-square tests unless otherwise indicated.

^a^ only among those who exchanged sex for goods/money (n = 84)

Mos–month

Yrs–years

Based on the multivariable model, age was the only socio-demographic characteristic associated with HIV seropositivity; older participants (25–35 years old) were more likely to be HIV positive (OR: 6,13, 95% CI: 3.01,12.5). The behavioral and biological factors that were significantly associated with HIV infection were: being MSM (OR: 9.07, 95% CI: 3.85, 21.4), self-perception of being at high-risk for HIV (OR: 3.99, 95% CI: 1.27, 12.5) and self-reporting a diagnosis of an STI in the last three months (OR: 3.75, 95% CI: 1.57, 8.97).

## Discussion

To our knowledge, this is the first HIV study to recruit general population and high-risk groups using a community-based strategy in Maputo city, Mozambique. The prevalence of HIV varies among different groups within our study, with MSM representing a higher proportion compared to the overall study population. Considering the small sample of MSM in the study it is not possible to compare with the results from the previous national surveys where HIV prevalence among MSM (N = 496) was lower (8.2%) [4] when compared to the overall HIV prevalence of 16.8% in Maputo City [11].

We also found that having a history of a known HIV positive partner, being MSM and perceiving oneself to be at high-risk were associated with HIV prevalence. Older age and no use of condoms were also associated with HIV prevalence, which is consistent with the findings from the baseline data described from HIV cohorts of women in other provinces of Mozambique [12]. We did not find an association between the number of sexual partners and HIV prevalence, which is consistent with the finding from a cohort study with low risk youths in Maputo City [9]; this may be due to the fact that not all reported sexual partners were concurrent. Similar to a cohort study conducted in Beira city [12], we did not find an association between exchanging sex for money, goods or favors and risk of acquiring HIV infection. This might also be related to the fact that most of the participants do not perform sex work as an occupation and/or do not disclose their real occupation. Although there is an association between no use of condom and HIV prevalence in the general study population, frequency of condom use with clients was not found to be associated with HIV prevalence within FSW, which is consistent with the findings from the FSW national survey in Mozambique [3].

We recognize that the prevalence found in this cross-sectional analysis was lower than the prevalence found in population-based surveys. This finding was is not surprising considering that one of the pre-eligibility criteria described on the fliers during recruitment was willingness to perform HIV tests. Individuals already aware of their positive HIV status were probably less likely to participate in the study, which can be confirmed as only 1.2% (13/1117) of those who presented to our clinic knew their HIV positive status. Moreover, the number of MSM, FSW and other high-risk groups screened for HIV was lower than expected, the low risk participants may have contributed to the lower overall HIV prevalence. Determining HIV prevalence was not the primary objective of the main incidence study; therefore, we were unable to control the bias on selecting the volunteers for the study.

A few limitations from our study can be noted. Firstly, our study identified male participants that exchanged sex for goods or money, however, the questionnaire did not further distinguish between those who bought or sold sex, which was a missed opportunity to potentially identify male sex workers, in particular, female transgender sex workers–a group with high burden of HIV [13, 14]. Secondly, although the LGBT and the FSW associations actively participated in the recruitment process, we recognize that we identified only a limited number of MSM and FSW in our study–previous MSM and FSW National Surveys estimated a population size of more than 10000 MSM and more than 13500 FSW. A factor that might have contributed to a lower participation of this population could be related to the recruitment method, as the Respondent Driven Sampling (RDS) method often used to identify hidden populations in high-risk studies [4, 12–17] was not applied. In addition, face-to-face interviews with unknown study staff might have discouraged the MSM and FSW study participants from disclosing their sexual behaviors. Use of Audio Computer-Assisted Self-Interview (ACASI) to ensure reliability of self-reported data for HIV prevention studies in Mozambique should have been considered. A study from Kenya reported that FSW were more likely to report high-risk behavior using ACASI compared to face-to-face interviews [18].

Despite these limitations, this study is the first cohort study in Mozambique to target these populations and high-risk individuals and recruit using community-based strategies. It is key to supporting Mozambique and INS in its first Phase III HIV efficacy trial, slated to start in 2018. The study highlights increased prevalence of HIV in key populations such as MSM and FSW in Maputo City, Mozambique and demonstrates that strategies to increase the willingness and access to HIV testing and prevention modalities in Maputo City are needed. Additionally, collaborating with organizations that focus on high-risk populations is critical to ensure access to these key populations for future vaccine efficacy and prevention studies and the success of Mozambique in the future HIV vaccine efficacy trial arena.

## Supporting information

S1 File Case report forms. (PDF)

## References

[1] UNAIDS. AIDS by the numbers 2015 http://www.unaids.org/en/resources/documents/2015/AIDS_by_the_numbers_20152015 [cited 2017 21 February].

[2] Instituto Nacional de Saúde INdEI, ICF Internacional. Inquérito de Indicadores de Imunização, Malária e HIV/SIDA em Moçambique 2015. Relatório Preliminar de Indicadores de HIV Maputo, Moçambique2015. Available from: https://www.researchgate.net/profile/Joel_Christian_Reed/publication/316629171_HIVAIDS_Key_Indicator_Report_Mozambique_IMASIDA_2015/links/59089ab2a6fdcc4961630277/HIV-AIDS-Key-Indicator-Report-Mozambique-IMASIDA-2015.pdf.

[3] Pond MJ, Nori AV, Witney AA, Lopeman RC, Butcher PD, Sadiq ST. High prevalence of antibiotic-resistant Mycoplasma genitalium in nongonococcal urethritis: the need for routine testing and the inadequacy of current treatment options. Clin Infect Dis. 2014;58(5):631–7. 10.1093/cid/cit752 24280088 PMC3922211

[4] Nala R, Cummings B, Horth R, Inguane C, Benedetti M, Chissano M, et al Men who have sex with men in Mozambique: identifying a hidden population at high-risk for HIV. AIDS Behav. 2015;19(2):393–404. Epub 2014/09/23. 10.1007/s10461-014-0895-8 25234252 PMC4341016

[5] Fauci AS, Marston HD. Ending AIDS—is an HIV vaccine necessary? N Engl J Med. 2014;370(6):495–8. Epub 2014/02/07. 10.1056/NEJMp1313771 . 24499210

[6] Bos JM, Postma MJ. The economics of HIV vaccines: projecting the impact of HIV vaccination of infants in sub-Saharan Africa. PharmacoEconomics. 2001;19(9):937–46. Epub 2001/11/10. 10.2165/00019053-200119090-00005 . 11700780

[7] Adamson B, Dimitrov D, Devine B, Barnabas R. The Potential Cost-Effectiveness of HIV Vaccines: A Systematic Review. PharmacoEconomics—open. 2017;1(1):1–12. Epub 2017/04/04. 10.1007/s41669-016-0009-9 28367539 PMC5373805

[8] Viegas EO, Tembe N, Nilsson C, Meggi B, Maueia C, Augusto O, et al Intradermal HIV-1 DNA Immunization Using Needle-Free Zetajet Injection Followed by HIV-Modified Vaccinia Virus Ankara Vaccination Is Safe and Immunogenic in Mozambican Young Adults: A Phase I Randomized Controlled Trial. AIDS research and human retroviruses. 2017 Epub 2017/10/04. 10.1089/aid.2017.0121 . 28969431 PMC6913121

[9] Viegas EO, Tembe N, Macovela E, Goncalves E, Augusto O, Ismael N, et al Incidence of HIV and the prevalence of HIV, hepatitis B and syphilis among youths in Maputo, Mozambique: a cohort study. PloS one. 2015;10(3):e0121452 Epub 2015/03/24. 10.1371/journal.pone.0121452 25798607 PMC4370560

[10] Republica de Mocambique MdS. Guia para Tratamento e Controle das Infecções de Transmissão Sexual. Maputo, Mozambique: 2006.

[11] Inquérito Nacional de Prevalência, Riscos Comportamentais e Informação sobre o HIV e SIDA em Moçambique https://dhsprogram.com/pubs/pdf/ais8/ais8.pdf: Ministério da Saúde Instituto Nacional de Saúde 2009 [cited 2017 21 February].

[12] Zango A, Dube K, Kelbert S, Meque I, Cumbe F, Chen PL, et al Determinants of prevalent HIV infection and late HIV diagnosis among young women with two or more sexual partners in Beira, Mozambique. PloS one. 2013;8(5):e63427 Epub 2013/05/22. 10.1371/journal.pone.0063427 23691046 PMC3656941

[13] Logie CH, Wang Y, Lacombe-Duncan A, Jones N, Ahmed U, Levermore K, et al Factors associated with sex work involvement among transgender women in Jamaica: a cross-sectional study. J Int AIDS Soc. 2017;20(1):21422 Epub 2017/04/14. 10.7448/IAS.20.01/21422 28406598 PMC5515035

[14] Cai Y, Wang Z, Lau JT, Li J, Ma T, Liu Y. Prevalence and associated factors of condomless receptive anal intercourse with male clients among transgender women sex workers in Shenyang, China. J Int AIDS Soc. 2016;19(3 Suppl 2):20800 Epub 2016/07/20. 10.7448/ias.19.3.20800 27431471 PMC4949316

[15] Dube K, Zango A, van de Wijgert J, Meque I, Ferro JJ, Cumbe F, et al HIV incidence in a cohort of women at higher risk in Beira, Mozambique: prospective study 2009–2012. PloS one. 2014;9(1):e84979 Epub 2014/01/30. 10.1371/journal.pone.0084979 24475035 PMC3903474

[16] Larmarange J, Wade AS, Diop AK, Diop O, Gueye K, Marra A, et al Men who have sex with men (MSM) and factors associated with not using a condom at last sexual intercourse with a man and with a woman in Senegal. PloS one. 2010;5(10). Epub 2010/10/20. 10.1371/journal.pone.0013189 20957157 PMC2950158

[17] Inguane C, Horth RZ, Miranda AE, Young PW, Sathane I, Cummings BE, et al Socio-demographic, Behavioral and Health Characteristics of Underage Female Sex Workers in Mozambique: The Need to Protect a Generation from HIV Risk. AIDS Behav. 2015;19(12):2184–93. Epub 2015/05/02. 10.1007/s10461-015-1068-0 25931241 PMC4776321

[18] van der Elst EM, Okuku HS, Nakamya P, Muhaari A, Davies A, McClelland RS, et al Is audio computer-assisted self-interview (ACASI) useful in risk behaviour assessment of female and male sex workers, Mombasa, Kenya? PloS one. 2009;4(5):e5340 Epub 2009/05/05. 10.1371/journal.pone.0005340 19412535 PMC2671594

