# Supplementary material for: HIV prevalence and risk behavior among male and female adults screened for enrolment into a vaccine preparedness study in Maputo, Mozambique
Source: PLoS One. 2019 Sep 17;14(9):e0221682. doi: 10.1371/journal.pone.0221682 (PMC6748437; doi:10.1371/journal.pone.0221682)
Supplement: S1 File — (PDF) [file pone.0221682.s001.pdf]

Site	RV-363	Cohort Development	Visit:
☐ 1 *CISPOC*<br>☐ 2 *CIDI*	Subject Number ___ ___ ___ ___ ___ ___		________

Date of Visit
DD/MON/YYYY __ __/__ __ __/__ __ __ __

ELIGIBILITY

			Yes	No*	Initials ID
1.	Is the subject HIV negative at screening?		☐ 1	☐ 0	☐☐☐
2.	Is the subject between the ages of 18-35 years old (inclusive)?		☐ 1	☐ 0	☐☐☐
3.	Does the subject report two or more sexual partners in the last 3 months?		☐ 1	☐ 0	☐☐☐
4.	Did the subject provide an Informed Consent?		☐ 1	☐ 0	☐☐☐
5.	Does the subject understand Portuguese?		☐ 1	☐ 0	☐☐☐
6.	Did the subject pass TOU (80% after 3 attempts)?<br>Score #1 ________ Score #2 ________ Score #3 ________		☐ 1	☐ 0	☐☐☐
7.	Is the subject available for the next 24 months to complete study visits?		☐ 1	☐ 0	☐☐☐
8.	Has the subject provided his/her contact information?		☐ 1	☐ 0	☐☐☐
9.	Has the subject provided contact information for one alternative personal contact?		☐ 1	☐ 0	☐☐☐
10.	Is the subject free of any preexisting medical, psychological, or social condition that would interfere with study participation?		☐ 1	☐ 0	☐☐☐
11.	Subject is NOT participating and has NOT participated in another HIV prevention study?		☐ 1	☐ 0	☐☐☐
12.	If the subject is female, is her pregnancy test negative?	**if Male**<br>☐ 8 N/A	☐ 1	☐ 0	☐☐☐

* *If any check box is marked "**NO**" (**other than #1**), the subject is **NOT ELIGIBLE** for enrollment into the study.*
*If #**1** is checked "**No**" then #**13** must be checked "**Yes**", otherwise the subject is **NOT ELIGIBLE**.*

13.	If #1 is checked "**No**", is subject being enrolled for masking purposes?	**if #1 is 'Yes'**<br>☐ 8 N/A	☐ 1	☐ 0	☐☐☐
14.	Is subject **ELIGIBLE** to participate in the study?		☐ 1	☐ 0	☐☐☐

________________	________________	__ __/__ __ __/__ __ __ __
**PI/Designee Print Name:**	**PI/Designee Signature:**	**Date Completed:** *DD/MON/YYYY*

QA Initial/Date ________________

1st Data Entry Initials/Date ________________

2nd Data Entry Initials/Date ________________

CONFIDENTIAL: This material is the property of WRAIR, and may not be disclosed or used except as authorized in writing by WRAIR.

Site	RV-363	Cohort Development	Visit:
☐ 1 ***CISPOC*** ☐ 2 ***CIDI***	Subject Number ___ ___ ___ ___ ___ ___		____________

Date of Visit
DD/MON/YYYY __ __/__ __ __/__ __ __ __

Demographic

1.	Gender: ☐ 0 Male ☐ 1 Female
2.	Date of Birth: __ __/__ __ __/1 9 __ __ *DD/MON/YYYY* Age: ☐☐ years old
3.	Place of Birth: ____________ *Country* ____________ *City(Village)* ____________ *Province*
4.	Highest level of education:<br>☐ 0 None<br>☐ 1 Primary school, not complete<br>☐ 2 Primary school, complete<br>☐ 3 Secondary school, not complete<br>☐ 4 Secondary school, complete<br>☐ 5 Attended or attending college or university
5.	Approximate monthly income (in meticais):<br>☐ 0 None<br>☐ 1 < 2,500.00 MT<br>☐ 2 2,501.00 – 5,000.00 MT<br>☐ 3 5,001.00 – 10,000.00 MT<br>☐ 4 10,001.00 MT – 20,000 MT<br>☐ 5 > 20,000 MT

Form Completed By: ____ ____ ____ **Date Completed:** __ __/__ __ __/__ __ __ __
DD/MON/YYYY

QA Initial/Date ______________________

1st Data Entry Initials/Date ______________________

2nd Data Entry Initials/Date ______________________

CONFIDENTIAL: This material is the property of WRAIR, and may not be disclosed or used except as authorized in writing by WRAIR.

Screening Medical History page 1 of 2

Site	RV-363	Cohort Development	Visit:
☐ 1 *CISPOC*<br>☐ 2 *CIDI*	Subject Number ___ ___ ___ ___ ___ ___		________

Date of Visit
DD/MON/YYYY __ __/__ __ __/__ __ __ __

Screening Medical History

1.	Has ever received a blood transfusion	☐ 0 No<br>☐ 1 Yes	☐ 88 Refused To Answer<br>☐ 99 Do Not Know
2.	Has been tested for HIV<br>***(* If "No" → skip to Q #5)***	☐ 0 No *<br>☐ 1 Yes	☐ 88 Refused To Answer<br>☐ 99 Do Not Know
3.	The last HIV test was done	☐ 1 Less than 6 months ago<br>☐ 2 Between 6 months and 12 months ago<br>☐ 3 More than 1 year ago	☐ 88 Refused To Answer<br>☐ 99 Do Not Know
4.	The result of the last HIV test was	☐ 0 Negative<br>☐ 1 Positive	☐ 88 Refused To Answer<br>☐ 99 Do Not Know
5.	Has been tested for Hepatitis<br>***(* If "No" → skip to Q #9)***	☐ 0 No *<br>☐ 1 Yes	☐ 88 Refused To Answer<br>☐ 99 Do Not Know
6.	The last Hepatitis test was done	☐ 1 Less than 6 months ago<br>☐ 2 Between 6 months and 12 months ago<br>☐ 3 More than 1 year ago	☐ 88 Refused To Answer<br>☐ 99 Do Not Know
7.	The result of the last Hepatitis test was<br>***(* If "Negative" → Skip to Q #9)***	☐ 0 Negative *<br>☐ 1 Positive	☐ 88 Refused To Answer<br>☐ 99 Do Not Know
8.	The type of Hepatitis was	☐ 1 Hepatitis B<br>☐ 2 Hepatitis C	☐ 88 Refused To Answer<br>☐ 99 Do Not Know
9.	Has ever been tested for TB<br>***(* If "No" → Skip to Q #12)***	☐ 0 No *<br>☐ 1 Yes	☐ 88 Refused To Answer<br>☐ 99 Do Not Know

QA Initial/Date ______________ 1st Data Entry Initials/Date ______________ 2nd Data Entry Initials/Date ______________

CONFIDENTIAL: This material is the property of WRAIR, and may not be disclosed or used except as authorized in writing by WRAIR.

Screening Medical History page 2 of 2

Site	*RV-363*	*Cohort Development*	Visit:
☐ 1 *CISPOC* ☐ 2 *CIDI*	Subject Number ___ ___ ___ ___ ___ ___		______

Date of Visit
DD/MON/YYYY __ __/__ __ __/__ __ __ __

Screening Medical History

10.	The result of the last TB test was<br>***(* If "Negative" → Skip to Q #12)***	☐ 0 Negative *<br>☐ 1 Positive	☐ 88 Refused To Answer<br>☐ 99 Do Not Know
11.	If **positive**, subject has been treated for TB previously or at this visit?	☐ 0 No<br>☐ 1 Yes	☐ 88 Refused To Answer<br>☐ 99 Do Not Know
12.	Has been diagnosed with Sexually Transmitted Infection (**STI**), in the **past 3 months**	☐ 0 No<br>☐ 1 Yes	☐ 88 Refused To Answer<br>☐ 99 Do Not Know
13.	Has been diagnosed with **malaria** in the **past 3 months**	☐ 0 No<br>☐ 1 Yes	☐ 88 Refused To Answer<br>☐ 99 Do Not Know
14.	Has a history of alcohol abuse	☐ 0 No<br>☐ 1 Yes	☐ 88 Refused To Answer<br>☐ 99 Do Not Know
15.	Has a history of non-injectable drug abuse	☐ 0 No<br>☐ 1 Yes	☐ 88 Refused To Answer<br>☐ 99 Do Not Know
16.	Has a history of injecting drug use<br>***(* If "No" → skip to Q #18)***	☐ 0 No *<br>☐ 1 Yes	☐ 88 Refused To Answer<br>☐ 99 Do Not Know
17.	Has a history of sharing needles	☐ 0 No<br>☐ 1 Yes	☐ 88 Refused To Answer<br>☐ 99 Do Not Know
18.	The age at first sexual intercourse	☐☐ years<br>Age:	☐ 88 Refused To Answer<br>☐ 99 Do Not Know

Form Completed By: ____ ____ ____

Date Completed: __ __/__ __ __/__ __ __ __
DD/MON/YYYY

QA Initial/Date ______________________

1st Data Entry Initials/Date ______________________

2nd Data Entry Initials/Date ______________________

CONFIDENTIAL: This material is the property of WRAIR, and may not be disclosed or used except as authorized in writing by WRAIR.

Site	RV-363	Cohort Development	Visit:
☐ 1 *CISPOC*<br>☐ 2 *CIDI*	Subject Number ___ ___ ___ ___ ___ ___		___________

Date of Visit
DD/MON/YYYY __ __/__ __ __/__ __ __ __

Screening Male Medical History

1.	Has been circumcised<br>(* ***If "No" → Skip to Q #4***)	☐ 0 No *<br>☐ 1 Yes	☐ 88 Refused To Answer<br>☐ 99 Do Not Know
2.	Subject age the day of circumcision<br>***Years of Age*** ☐☐ OR		☐ 1 Neonatal Circumcision<br>☐ 88 Refused To Answer<br>☐ 99 Do Not Know
3.	Where was the circumcision done	☐ 1 Hospital<br>☐ 2 Traditional	☐ 88 Refused To Answer<br>☐ 99 Do Not Know
4.	Was the subject referred to counseling for male circumcision education and procedures at this visit?<br>******** *Ask this question* ***only*** *if question #* ***1*** *answer is* ***"No"***	☐ 0 No<br>☐ 1 Yes	
5.	Number of sexual partners in the **last 3 months**: ☐☐☐		
6.	How many of these sexual partners were concurrent? ☐☐☐		

Form Completed By: ____ ____ ____ **Date Completed:** __ __/__ __ __/__ __ __ __
DD/MON/YYYY

QA Initial/Date ____________ 1st Data Entry Initials/Date ____________ 2nd Data Entry Initials/Date ____________

CONFIDENTIAL: This material is the property of WRAIR, and may not be disclosed or used except as authorized in writing by WRAIR.

Site	RV-363	Cohort Development	Visit:
☐ 1 CISPOC<br>☐ 2 CIDI	Subject Number ___ ___ ___ ___ ___ ___		___________

Date of Visit DD/MON/YYYY __ __/__ __ __/__ __ __ __

Screening Female Medical History

1.	Has received reproductive tract surgery, including tubal sterilization<br>* If "Yes", specify:________________	☐ 0 No<br>☐ 1 Yes *	☐ 88 Refused To Answer<br>☐ 99 Do Not Know
2.	Has had history of irregular menses<br>* If "Yes", specify:________________	☐ 0 No<br>☐ 1 Yes *	☐ 88 Refused To Answer<br>☐ 99 Do Not Know
3.	Has had any gynecological problems<br>* If "Yes", specify:________________	☐ 0 No<br>☐ 1 Yes *	☐ 88 Refused To Answer<br>☐ 99 Do Not Know
4.	Current desire to get pregnant in the next year	☐ 0 No<br>☐ 1 Yes<br>☐ 2 Maybe	☐ 8 Not Applicable<br>☐ 88 Refused To Answer<br>☐ 99 Do Not Know
5.	Number of sexual partners in the **<u>last 3 months</u>**:	☐☐☐	
6.	How many of these sexual partners were concurrent?	☐☐☐	

Form Completed By: ____ ____ ____ **Date Completed:** DD/MON/YYYY __ __/__ __ __/__ __ __ __

QA Initial/Date ________________

1st Data Entry Initials/Date ________________

2nd Data Entry Initials/Date ________________

CONFIDENTIAL: This material is the property of WRAIR, and may not be disclosed or used except as authorized in writing by WRAIR.

Site	RV-363	Cohort Development	Visit:
☐ 1 *CISPOC* ☐ 2 *CIDI*	Subject Number ___ ___ ___ ___ ___ ___		____________

Date of Visit *DD/MON/YYYY* _ _/_ _ _/_ _ _ _   ☐ Missed Visit ☐ Not Done

Medical History – Interim Visits and Exit Visit

1.	Was subject referred for follow-up medical evaluation(s) for **STI** or **Malaria** or **both** at the previous study visit? ☐ 0 No * ☐ 1 Yes ***(* If "No" → skip to Q #4)***
2.	If subject was referred, was he/she diagnosed with: **STI:** → ☐ 0 No ☐ 1 Yes * ***(* If "Yes" → is it)*** ☐ 1 Documented Report ☐ 2 Per Subject Report **Malaria:** → ☐ 0 No ☐ 1 Yes * ***(* If "Yes" → is it)*** ☐ 1 Documented Report ☐ 2 Per Subject Report
3.	Did subject receive treatment for: ☐ STI → ☐ 1 Documented Report ☐ 2 Per Subject Report ☐ Malaria → ☐ 1 Documented Report ☐ 2 Per Subject Report ☐ Neither

QA Initial/Date ____________________

1st Data Entry Initials/Date ____________________

2nd Data Entry Initials/Date ____________________

CONFIDENTIAL: This material is the property of WRAIR, and may not be disclosed or used except as authorized in writing by WRAIR.

Site	RV-363	Cohort Development	Visit:
☐ 1 CISPOC<br>☐ 2 CIDI	Subject Number ___ ___ ___ ___ ___ ___		___________

Date of Visit DD/MON/YYYY __ __/__ __ __/__ __ __ __ ☐ Missed Visit ☐ Not Done

Medical History – Interim Visits and Exit Visit

4. At this visit or in the last 3 months, subject has experienced the following symptoms of **STI**?

(Check all that apply)

- ☐ None
- ☐ Penile/vaginal discharge
- ☐ Lower abdominal pain
- ☐ Penile/vaginal itching or burning
- ☐ Pain during intercourse
- ☐ Rectal pain or pain with defecation
- ☐ Rectal discharge
- ☐ Rectal bleeding
- ☐ Genital ulceration
- ☐ Dysuria
- ☐ Genital condylomata
- ☐ Unknown
- ☐ Other, specify________________________

5. At this visit or in the last 3 months, subject has experienced the following symptoms of **malaria**?

(Check all that apply)

- ☐ None
- ☐ Fever
- ☐ Chills
- ☐ Headache
- ☐ Sweats
- ☐ Fatigue
- ☐ Nausea or vomiting
- ☐ Unknown
- ☐ Other, specify ________________________

Form Completed By: ____ ____ ____ **Date Completed:** DD/MON/YYYY __ __/__ __ __/__ __ __ __

QA Initial/Date ______________ 1st Data Entry Initials/Date ______________ 2nd Data Entry Initials/Date ______________

CONFIDENTIAL: This material is the property of WRAIR, and may not be disclosed or used except as authorized in writing by WRAIR.

Vital Signs/Physical Exam

Site	RV-363	Cohort Development	Visit:
☐ 1 *CISPOC* ☐ 2 *CIDI*	Subject Number ___ ___ ___ ___ ___ ___		______________

Date of Visit *DD/MON/YYYY* __ __/__ __ __/__ __ __ __

☐ Missed Visit
☐ Not Done

Vital Signs

Completed By: ___ ___ ___ **Date Completed:** __ __ /__ __ __ /__ __ __ __ (*DD MON YYYY*)

**Height** *(cm)*	**Weight** *(kg)*	**Temperature** *(˚C)* ☐ 1 *Oral* ☐ 2 *Axillary*	**SITTING MEASUREMENTS**			
			**Pulse** *(beats/min)*	**Respirations** *(breaths/min)*	**Systolic BP** *(mmHg)*	**Diastolic BP** *(mmHg)*
________	________	________	________	________	________	________

Physical Examination

☐ **Complete** ☐ **Targeted**

Examine the following and check appropriate box for each body system

**BODY SYSTEM**	**Normal**	***Abnormal**	**Not Done**	*** ONLY COMMENT ON ABNORMALITIES**
General Appearance	☐ 0	☐ 1	☐ 9	
Skin	☐ 0	☐ 1	☐ 9	
HEENT	☐ 0	☐ 1	☐ 9	
Lymphatic	☐ 0	☐ 1	☐ 9	
Pulmonary	☐ 0	☐ 1	☐ 9	
Cardiovascular	☐ 0	☐ 1	☐ 9	
Abdominal	☐ 0	☐ 1	☐ 9	
Genitourinary	☐ 0	☐ 1	☐ 9	
Musculo-skeletal	☐ 0	☐ 1	☐ 9	
Psychiatric	☐ 0	☐ 1	☐ 9	
Neurologic	☐ 0	☐ 1	☐ 9	
Other, *specify* ______________	☐ 0	☐ 1	☐ 9	

Respond to the question below at Screening visit only:

Is subject free of any medical condition that would preclude their study participation? ☐ 0 No ☐ 1 Yes

Form Completed By: ____ ____ ____ **Date Completed:** *DD/MON/YYYY* __ __/__ __ __/__ __ __ __

QA Initial/Date ______________
1st Data Entry Initials/Date ______________
2nd Data Entry Initials/Date ______________

CONFIDENTIAL: This material is the property of WRAIR, and may not be disclosed or used except as authorized in writing by WRAIR.

Medical Assessment

MDA

Site	RV-363	Cohort Development	Visit:
☐ 1 *CISPOC* ☐ 2 *CIDI*	Subject Number ___ ___ ___ ___ ___ ___		________

Date of Visit *DD/MON/YYYY* __ __/__ __ __/__ __ __ __   ☐ Missed Visit ☐ Not Done

Medical Assessment

1.	Was subject referred for an **STI** evaluation test at this visit?	☐ 0 No ☐ 1 Yes
2.	Was subject provided a prescription for an **STI** at this visit?	☐ 0 No ☐ 1 Yes
3.	Was the subject referred for **malaria** evaluation at this visit?	☐ 0 No ☐ 1 Yes
4.	Was a **malaria** test completed at this visit? ***(* If "Yes" complete Malaria Lab Test)***	☐ 0 No ☐ 1 Yes *
5.	Was the subject provided a prescription for **malaria** at this visit?	☐ 0 No ☐ 1 Yes
6.	Did subject report any **social** problem because he/she is in the study? ***(* If "Yes" complete SHE CRF)***	☐ 0 No ☐ 1 Yes * ☐ 8 Not Applicable - ***(must be checked at screening only)***
7.	Was **Pregnancy Test** completed? ***(* If "Yes" complete below)***	☐ 0 No, specify reason: ____________ ☐ 1 Yes * ☐ 8 Not Applicable, Male
	**Date of Sample** *DD/MON/YYYY* __ __/__ __ __/__ __ __ __	
	Pregnancy result:	☐ 0 Negative ☐ 1 Positive

Form Completed By: ____ ____ ____   **Date Completed:** *DD/MON/YYYY* __ __/__ __ __/__ __ __ __

QA Initial/Date ____________   1st Data Entry Initials/Date ____________   2nd Data Entry Initials/Date ____________

CONFIDENTIAL: This material is the property of WRAIR, and may not be disclosed or used except as authorized in writing by WRAIR.

**Site**<br>☐ 1 ***CISPOC***<br>☐ 2 ***CIDI***	***RV-363***	***Cohort Development***<br>**Subject Number** ___ ___ ___ ___ ___ ___	**Visit:**<br>__________

Date of Visit *DD/MON/YYYY* _ _/_ _ _/_ _ _ _

☐ Missed Visit
☐ Not Done

HIV Testing

☐ Not Applicable, **Previously Seroconverted**

Date of Sample *DD/MON/YYYY* _ _/_ _ _/_ _ _ _

**Test**		**Value**
1.	Determine HIV Rapid Test	☐ 0 Non-Reactive<br>☐ 1 Reactive<br>☐ 9 Not Done
2.	Unigold HIV Test<br><br>*** If discordant from Determine do Elisa*	☐ 0 Non-Reactive<br>☐ 1 Reactive<br>☐ 2 Not Required<br>☐ 9 Not Done
3.	ELISA	☐ 0 Non-Reactive<br>☐ 1 Reactive<br>☐ 2 Not Required<br>☐ 9 Not Done

Form Completed By: ____ ____ ____

Date Completed: *DD/MON/YYYY* _ _/_ _ _/_ _ _ _

QA Initial/Date ____________

1st Data Entry Initials/Date ____________

2nd Data Entry Initials/Date ____________

CONFIDENTIAL: This material is the property of WRAIR, and may not be disclosed or used except as authorized in writing by WRAIR.

HIV Viral Burden

HVB

**Site**	***RV-363***	***Cohort Development***	**Visit:**
☐ 1 ***CISPOC*** ☐ 2 ***CIDI***	**Subject Number** ___ ___ ___ ___ ___ ___		______________

Date of Visit *DD/MON/YYYY* __ __/__ __ __/__ __ __ __ ☐ Missed Visit ☐ Not Done

Date of Sample *DD/MON/YYYY* __ __/__ __ __/__ __ __ __

HIV Viral Burden

☐ 0 Not Detected

☐ 1 Detected, < 20 copies/mL

☐ 2 Equal to ______________ copies/mL

☐ 3 > 10.000.000 copies/mL

Form Completed By: ____ ____ ____ **Date Completed:** *DD/MON/YYYY* __ __/__ __ __/__ __ __ __

QA Initial/Date ______________________

1st Data Entry Initials/Date ______________________

2nd Data Entry Initials/Date ______________________

CONFIDENTIAL: This material is the property of WRAIR, and may not be disclosed or used except as authorized in writing by WRAIR.

Site	RV-363	Cohort Development	Visit
☐ 1 *CISPOC* ☐ 2 *CIDI*	Subject Number ___ ___ ___ ___ ___ ___		________

Date of Visit *DD/MON/YYYY* __ __/__ __ __/__ __ __ __ ☐ Missed Visit ☐ Not Done

Instruction:- '**Not Applicable**' check box to be used only if those specific Lymphocytes test(s) will **not** be complete at **your site**

Date of Sample *DD/MON/YYYY* __ __/__ __ __/__ __ __ __

Lymphocytes

CD3 + T-Lymphocyte (cells/ul) ____________ ☐ Not Done

CD3 + T-Lymphocyte (%) ____________ ☐ Not Done ☐ Not Applicable

CD3 + CD4 + Helper T-Lymphocyte (cells/ul) ____________ ☐ Not Done

CD3 + CD4 + Helper T-Lymphocyte (%) ____________ ☐ Not Done ☐ Not Applicable

CD3 + CD8 Suppressor T-Lymphocyte (cells/ul) ____________ ☐ Not Done ☐ Not Applicable

CD3 + CD8 Suppressor T-Lymphocyte (%) ____________ ☐ Not Done ☐ Not Applicable

Form Completed By: ____ ____ ____ **Date Completed:** *DD/MON/YYYY* __ __/__ __ __/__ __ __ __

QA Initial/Date ____________ 1st Data Entry Initials/Date ____________ 2nd Data Entry Initials/Date ____________

CONFIDENTIAL: This material is the property of WRAIR, and may not be disclosed or used except as authorized in writing by WRAIR.

Site	RV-363	Cohort Development	Visit:
☐ 1 *CISPOC*<br>☐ 2 *CIDI*	Subject Number ___ ___ ___ ___ ___ ___		___________

Date of Visit *DD/MON/YYYY* __ __/__ __ __/__ __ __ __ ☐ Not Done

Instruction:- '**Not Applicable (NA)**' check box to be used only if those specific Differential test(s) will **not** be complete at **your site**

Date of Sample *DD/MON/YYYY* __ __/__ __ __/__ __ __ __

Hematology

WBC (Thous/µL) ____________________

RBC (MILL/µL) ____________________

HGB (G/DL) ____________________

HCT (%) ____________________

MCV (fL) ____________________

MCH (pg) ____________________

MCHC (g/dL) ____________________

RDW-SD (fL) ____________________

RDW-CV (%) ____________________

PLT (Thous/µL) ____________________

MPV (fL) ____________________

Differential

☐ **Not Done**

NEUT (%) ____________

NEUT # (Thous/µL) ____________

LYMPH (%) ____________

LYMPH # (Thous/µL) ____________

MXD (%) ____________ ☐ NA

MXD # (Thous/µL) ____________ ☐ NA

MONO (%)____________ ☐ NA

MONO # (Thous/µL)____________ ☐ NA

EOS (%) ____________ ☐ NA

EOS # (Thous/µL) ____________ ☐ NA

BASO (%) ____________ ☐ NA

BASO # (Thous/µL) ____________ ☐ NA

Form Completed By: ____ ____ ____ **Date Completed:** *DD/MON/YYYY* __ __/__ __ __/__ __ __ __

QA Initial/Date ____________________

1st Data Entry Initials/Date ____________________

2nd Data Entry Initials/Date ____________________

CONFIDENTIAL: This material is the property of WRAIR, and may not be disclosed or used except as authorized in writing by WRAIR.

Site	RV-363	Cohort Development	Visit:
☐ 1 ***CISPOC*** ☐ 2 ***CIDI***	**Subject Number** ___ ___ ___ ___ ___ ___		____________

Date of Visit *DD/MON/YYYY* __ __/__ __ __/__ __ __ __ ☐ Not Done

Serum Chemistry

Date of Sample *DD/MON/YYYY* __ __/__ __ __/__ __ __ __

ALT/SGPT (U/L) ________________ ☐ Not Done

Creatinine ____________________ ☐ Not Done

☐ 1 umol/L ☐ 2 mg/dL

Total Bilirubin __________________ ☐ Not Done

☐ 1 umol/L ☐ 2 mg/dL

Direct Bilirubin _________________ ☐ Not Done

☐ 1 umol/L ☐ 2 mg/dL

Glucose ______________________ ☐ Not Done

☐ 1 mmol/L ☐ 2 mg/dL

Form Completed By: ____ ____ ____ **Date Completed:** *DD/MON/YYYY* __ __/__ __ __/__ __ __ __

QA Initial/Date	1st Data Entry Initials/Date	2nd Data Entry Initials/Date
______________________	______________________	______________________

CONFIDENTIAL: This material is the property of WRAIR, and may not be disclosed or used except as authorized in writing by WRAIR.

Syphilis Test SYP

Site	RV-363	Cohort Development	Visit:
☐ 1 ***CISPOC*** ☐ 2 ***CIDI***	**Subject Number** ___ ___ ___ ___ ___ ___		____________

Date of Visit *DD/MON/YYYY* __ __/__ __ __/__ __ __ __ ☐ Not Done

Syphilis Lab Test

Was syphilis serology done? ☐ 0 No ☐ 1 Yes *

*(* if **"Yes"** complete below)*

Date of Sample *DD/MON/YYYY* __ __/__ __ __/__ __ __ __

Syphilis Initial

RPR
- ☐ 0 Negative
- ☐ 1 Positive 1: ______________ titer
- ☐ 2 Indeterminate
- ☐ 9 Not Done

Confirmatory

TPPA
- ☐ 0 Negative
- ☐ 1 Positive
- ☐ 2 Indeterminate
- ☐ 8 Not Required
- ☐ 9 Not Done

Form Completed By: ____ ____ ____ **Date Completed:** *DD/MON/YYYY* __ __/__ __ __/__ __ __ __

QA Initial/Date ______________________

1st Data Entry Initials/Date ______________________

2nd Data Entry Initials/Date ______________________

CONFIDENTIAL: This material is the property of WRAIR, and may not be disclosed or used except as authorized in writing by WRAIR.

Site	RV-363	Cohort Development	Visit:
☐ 1 *CISPOC*<br>☐ 2 *CIDI*	**Subject Number** ___ ___ ___ ___ ___ ___		________

Date of Visit
DD/MON/YYYY __ __/__ __ __/__ __ __ __

Malaria Lab Test

Date of Sample
DD/MON/YYYY __ __/__ __ __/__ __ __ __

Instruction:- '**Not Applicable**' choice to be used only if some of the Malaria test(s) will **not** be complete at **your site**

1. Malaria rapid diagnostic test (RDT) result:

- ☐ 0 Negative
- ☐ 1 Plasmodium falciparum
- ☐ 2 Plasmodium vivax
- ☐ 3 Mixed infection
- ☐ 4 Equivocal or invalid
- ☐ 8 Not Applicable
- ☐ 9 Not Done

2. a) Smear Microscopy:

- ☐ 1 Plasmodium falciparum
- ☐ 2 Other, specify ____________________
- ☐ 8 Not Applicable
- ☐ 9 Not Done

b) Parasite density:

- ☐ 1 +
- ☐ 2 + +
- ☐ 3 + + +
- ☐ 4 + + + +
- ☐ 5 + + + + +
- ☐ 6 Indeterminate
- ☐ 8 Not Applicable

Form Completed By: ____ ____ ____ **Date Completed:** __ __/__ __ __/__ __ __ __
DD/MON/YYYY

QA Initial/Date ________ 1st Data Entry Initials/Date ________ 2nd Data Entry Initials/Date ________

CONFIDENTIAL: This material is the property of WRAIR, and may not be disclosed or used except as authorized in writing by WRAIR.

TERMINATION FROM STUDY

Site	RV-363	Cohort Development	Last Visit:
☐ 1 CISPOC<br>☐ 2 CIDI	Subject Number ___ ___ ___ ___ ___ ___		____________

Termination From Study

Date of Termination from Study _ _/_ _ _/_ _ _ _
DD/MON/YYYY

What was the last visit # completed by the subject? _________________

Reason for Termination

☐ 1 Completed the final scheduled study visit

☐ 2 Subject decision (subject has indicated that participation is terminated permanently and no further contact is permitted) ***(Check all that apply)***

- ☐ Pregnant does not want to continue
- ☐ Unable to continue keeping visits
- ☐ Significant social harm event
- ☐ HIV+ does not want to continue
- ☐ Moved from the area
- ☐ Incarceration
- ☐ Withdraw consent
- ☐ Other, specify ____________________

☐ 3 Death, complete additional information below, complete Death Report form and inform MHRP COO and Mozambique Ethics Committee immediately within 48 hours

Date of Death _ _/_ _ _/_ _ _ _
DD/MON/YYYY

Primary Cause of Death ☐ 0 Unknown ☐ 1 Other, specify ______________________

☐ 4 Non-compliance with visit schedule/Investigator decision

☐ 5 Protocol violation, specify ______________________________________

☐ 6 Concurrent illness/medication reason, specify ______________________________

☐ 7 Reaction to blood collection, specify ______________________________

☐ 8 Lost to follow-up

☐ 9 Other reason, specify ______________________________________

PI/Designee Print Name:	PI/Designee Signature	Date Completed: DD/MON/YYYY
____________________	____________________	_ _/_ _ _/_ _ _ _

QA Initial/Date ________________________

1st Data Entry Initials/Date ________________________

2nd Data Entry Initials/Date ________________________

CONFIDENTIAL: This material is the property of WRAIR, and may not be disclosed or used except as authorized in writing by WRAIR.

Consent Use of Specimens

CUS

Site	RV-363	Cohort Development	Visit:
☐ 1 CISPOC<br>☐ 2 CIDI	Subject Number ___ ___ ___ ___ ___ ___		________

Date of Visit
DD/MON/YYYY __ __/__ __ __/__ __ __ __

Instruction:- Please complete this CRF each time a participant is consented (i.e screening visit) and re-consented, as well as if there is a change in consent for the use of specimens.

Informed Consent Form (ICF) Version #: ________

Consent to use of Specimens

		No	Yes
1.	I agree to allow my samples to be used for the genetic testing as described for this study.	☐ 0	☐ 1
2.	I agree to the storage of my samples and to the future use of these samples in scientific studies as approved by the Ethics Review Boards.	☐ 0	☐ 1
3.	I agree to allow my samples to be used for genetic testing in future scientific studies as approved by the Ethics Review Boards	☐ 0	☐ 1
4.	I agree to allow my samples to be shipped to laboratories in other countries	☐ 0	☐ 1

Form Completed By: ____ ____ ____

Date Completed: __ __/__ __ __/__ __ __ __
DD/MON/YYYY

QA Initial/Date ______________________

1st Data Entry Initials/Date ______________________

2nd Data Entry Initials/Date ______________________

CONFIDENTIAL: This material is the property of WRAIR, and may not be disclosed or used except as authorized in writing by WRAIR.

CASE REPORT FORM REVIEW

CRR

Site	RV-363	Cohort Development	Visit:
☐ 1 CISPOC<br>☐ 2 CIDI	Subject Number ___ ___ ___ ___ ___ ___		________________

CRF REVIEW STATEMENT

I have reviewed all data contained in this case report form binder and verified that the contents are consistent with observations and source records. They accurately reflect the condition of the subject before, during and at the completion of the study.

Principal Investigator's/Designee **Date Completed :**

____ __ __/__ __ __/__ __ __ __

Name *Signature* *DD/MON/YYYY*

QA Initial/Date ______________________

1st Data Entry Initials/Date ______________________

2nd Data Entry Initials/Date ______________________

CONFIDENTIAL: This material is the property of WRAIR, and may not be disclosed or used except as authorized in writing by WRAIR.
